# Parent version of the Eating Disorder Examination: Reliability and validity in a treatment-seeking sample

**DOI:** 10.1186/s40337-024-01062-4

**Published:** 2024-07-18

**Authors:** Lisa Hail, Catherine R. Drury, Robert E. McGrath, Stuart B. Murray, Elizabeth K. Hughes, Susan M. Sawyer, Daniel Le Grange, Katharine L. Loeb

**Affiliations:** 1 Department of Psychiatry and Behavioral Sciences, University of California, San Francisco, UCSF Weill Institute for Neurosciences, 675 18th Street, San Francisco, CA USA; 2 School of Psychology and Counseling, Fairleigh Dickinson University, Teaneck, NJ USA; 3 Department of Psychiatry and the Behavioral Sciences, University of Southern California, Los Angeles, CA USA; 4 Department of Paediatrics, The University of Melbourne, Melbourne, Australia; 5 Murdoch Children’s Research Institute, Melbourne, Australia; 6 Department of Psychiatry and Behavioral Neuroscience (emeritus), The University of Chicago, Chicago, IL USA; 7 Chicago Center for Evidence-Based Treatment, Chicago, IL USA

**Keywords:** Eating disorder, Anorexia nervosa, Assessment, Adolescents, Parents

## Abstract

**Background:**

Assessment of eating disorders (ED) in youth relies heavily on self-report, yet persistent lack of recognition of the presence and/or seriousness of symptoms can be intrinsic to ED. This study examines the psychometric properties of a semi-structured interview, the parent version of the Eating Disorder Examination (PEDE), developed to systematically assess caregiver report of symptoms.

**Methods:**

A multi-site, clinical sample of youth (*N* = 522; age range: 12 to 18 years) seeking treatment for anorexia nervosa (AN) and subsyndromal AN were assessed using the Eating Disorder Examination (EDE) for youth and the PEDE for collateral caregiver report.

**Results:**

Internal consistencies of the four PEDE subscales were on par with established ranges for the EDE. Significant medium-sized correlations and poor to moderate levels of agreement were found between the corresponding subscales on each measure. For the PEDE, confirmatory factor analysis of the EDE four-factor model provided a poor fit; an exploratory factor analysis indicated that a 3-factor model better fits the PEDE.

**Conclusions:**

Findings suggest that the PEDE has psychometric properties on par with the original EDE. The addition of the caregiver perspective may provide incremental information that can aid in the assessment of AN in youth. Future research is warranted to establish psychometric properties of the PEDE in broader transdiagnostic ED samples.

## Abstract (plain language summary)

Assessments for eating disorders rely primarily on self-report; yet, the denial of symptoms or symptom severity among adolescents with anorexia nervosa can complicate assessment and delay treatment in this population. The Parent Eating Disorder Examination (PEDE) is the first semi-structured interview formally developed to improve childhood eating disorder assessment by including caregiver perspectives. In this study, a large sample of adolescents with anorexia nervosa completed a self-report interview (the Eating Disorder Examination or EDE) and their parents completed the PEDE. The PEDE appeared to measure parents’ report of their child’s eating disorder symptoms consistently. Results from both interviews were related to one another but did not completely agree. This suggests that in an eating disorder assessment, the PEDE can provide additional information from caregivers that might reduce diagnostic confusion and lead to earlier intervention for youth with anorexia nervosa.

## Background

With the publication of the fifth edition of the *Diagnostic and Statistical Manual of Mental Disorders* (*DSM-5*) [1], the criteria for diagnosing eating disorders (ED) were revised to reflect greater developmental sensitivity for youth. These modifications were particularly important as the onset of ED is most common in adolescence [2]. However, there remain many challenges to diagnosing restrictive ED, such as anorexia nervosa (AN), in children and adolescents, which could delay treatment of a pernicious, often refractory disorder.

One of the most significant challenges in relying on self-report in ED assessment and case identification is the persistent lack of recognition of the seriousness of symptoms, a core diagnostic feature of AN, which renders history of illness and present symptoms vulnerable to inaccuracies [3–5]. However, typical assessment methods for ED rely primarily on self-report, and may therefore be insufficient, particularly for younger individuals [6–9]. Compared to adults, adolescents generally score lower on measures of ED pathology despite similar levels of malnutrition [10], and appear to experience ED symptoms differently [11]. Minimization might be intrinsic to a developmentally normative limitation in recognizing the potential consequences of risky behaviors such as those associated with ED [5, 10, 12, 13]. Shorter duration of illness could compound this, further limiting adolescents’ appreciation of the current and future impact of what could in fact become a severe and enduring disorder [14, 15]. Relatedly, adolescents are unlikely to independently seek help for their ED, and may even engage in strategic minimization of symptoms, to avoid the implications of symptom endorsement (e.g., intervention efforts on the part of adults).

In addition, there are cognitive and emotional obstacles to evaluating symptoms of AN in youth, as several of the criteria are psychological in nature. For example, the ability to report a fear of weight gain requires that the young person be able to recognize and label their affective state correctly, and to identify the motivation behind their behavior [15, 16]. Other criteria are more abstract in nature (e.g., disturbance in the experience of shape and weight, undue influence of shape and weight on self-evaluation), and require the developmental maturation of abstract reasoning to recognize and endorse ED symptoms [2, 17–19].

The utility of multi-informant methods of assessing child psychopathology is long-established, and approaches have advanced over time [6, 20, 21]. However, most measures used for youth with ED – with notable exceptions [3] – rely exclusively on direct patient report [7] despite the unique risks posed by false negatives in case identification, particularly of AN. Two studies have examined parent-child concordance on the Eating Disorder Examination (EDE) [22] by administering the interview to parents with minimal modifications to the measure [18, 23]. For example, Couturier and colleagues [23] simply changed wording of questions from *you* to *your child* and retained items reflecting the internal experience of the child without prompting parents for data on why and how these experiences can be inferred through the child’s behavior. They found that youth with AN scored lower than their parents on two EDE subscales (Restraint and Weight Concern), while Mariano and colleagues [18] found good concordance between youth and parent scores. Mariano and colleagues [18] proposed that adolescents in their study were less likely to minimize their symptoms due to the timing of EDE administration (i.e., at the end of a two-day psychological assessment). It is also possible that more extensive adaptations to the EDE are needed to assist parents in consistently providing a comprehensive report of symptoms.

To address the need for a standardized method for including parental report in the assessment of ED, we developed a parent version of the EDE (PEDE) [7, 24, 36], with permission and input from the first author of the original measure [22], that mirrors the EDE but includes detailed questions to assess for observable indicators of ED. Although the EDE can be administered as young as 14 years and has been adapted for use in children aged 8 years and older [25], these assessments do not incorporate caregiver perspectives. Thus, the overall objective of the current study was to evaluate the psychometric properties of the PEDE in a large, multi-site sample of children and adolescents seeking treatment for AN and subsyndromal AN (SAN). Specifically, we examined the internal consistency of the PEDE subscales and the PEDE’s convergent and construct validity in relation to the EDE. We also aimed to compare PEDE and EDE rates of AN diagnosis. We hypothesized that:

1. Internal consistency of the four PEDE subscales (Restraint, Eating Concern, Weight Concern, Shape Concern) and global score as measured by Cronbach’s alpha reliability coefficients would be similar to those previously established for the EDE, which found values in the range from .44 to .85 [26].
2. Convergent validity would be demonstrated by small, positive correlations and low to moderate levels of agreement between the established EDE subscales and the corresponding subscales on the PEDE. These positive relationships would indicate that the subscales are tapping into similar constructs. The small effect sizes and low to moderate levels of agreement (as opposed to good or excellent) would suggest that both youth and parent perspectives offer incremental information to an ED assessment [23].
3. Both the EDE and PEDE would have a different factor structure than the four original subscales presented by Cooper and colleagues [27] as no studies evaluating factor structure in the EDE have confirmed this model. No specific hypotheses were possible for the expected factor structure given variations in the results of three prior studies [28–30], only one of which included adolescents [28].
4. The PEDE would yield a diagnosis of AN more frequently than the EDE among participants with both AN and SAN.

## Methods

### Participants

Participants were youth and guardian informants who presented to two research-based ED treatment programs in the United States (US; New York and Chicago) and one in Melbourne, Australia. Researchers at these sites received training on the EDE and PEDE, administered both interviews to youth and their caregivers presenting to clinical research centers for treatment of a suspected ED, and contributed deidentified baseline data as part of this multisite collaboration to establish the PEDE’s psychometric properties. Any larger studies [31, 32] from which these deidentified data were derived for secondary analyses were approved by the respective institutions’ institutional review boards; the present study was designated exempt from board review.

In order to assess the reliability and validity of the PEDE in a relatively homogenous sample, this study focused specifically on youth presenting to these sites with probable AN or SAN [32], a site-specific research category that would fall under other specified feeding and eating disorder (OSFED) in *DSM-5-TR* nomenclature. The original inspiration for developing the PEDE was to help identify true caseness in the context of underweight ED where denial and minimization are prominent and therefore parental report may be most useful [23]. Thus, submitted cases (*n =* 833) were excluded from analysis if one or more of the following were met: (a) percent expected body weight (EBW) based on median body mass index (mBMI) was greater than 100% (*n* = 232, 27.85%), (b) criteria for bulimia nervosa or binge eating disorder might be met by virtue of 12 or greater EDE objective bulimic episodes in the past three months and weight > 85% of EBW (*n* = 0), (c) age was younger than 12 years (*n* = 83, 9.96% of the full sample), or (d) there was insufficient information to accurately determine EBW (*n* = 1). Although low weight is a relative, personalized construct and population norms are not a valid benchmark against which to determine individual-level weight status, these weight criteria were used to reduce the likelihood of false positives and because not all sites recorded a more individualized measure of EBW and all reported percent of mBMI. The resulting sample included 522 youth paired with guardian informants, ranging in age from 12 to 18 years (*M* = 15.4; *SD* = 1.7), 89.7% parent- or self-identified as female, who were at 54–99% of mBMI (*M* = 84.3%; *SD* = 8.5). Further demographic data (including caregiver gender identity) were not reported consistently across all sites. The majority of participants were recruited from sites in Chicago (*n* = 219; 42.0%) and Melbourne (*n* = 260; 49.8%); 8.2% of participants (*n* = 43) were recruited from the New York-based site. There was a significant difference in PEDE global scores across sites (*F*(2,6) = 7.49, *p* = .002, η^2^ = 0.03), with guardians in New York reporting higher levels of ED pathology than those in Chicago or Melbourne (*p* = .002). EDE global scores did not significantly differ across sites (*p* = .725).

### Measures

#### Eating Disorder Examination (EDE) Version 16.0

The EDE [22] is a semi-structured clinical interview that was originally developed for use with adults but is also used, and has been found psychometrically acceptable, as a diagnostic and predictive tool with younger populations [33, 34]. The EDE is comprised of 33 items and uses a 7-point scale to measure the frequency (0 = “absence of the feature”; 6 = “feature present every day”) and severity (0 = “absence of the feature;” 6 = “feature present to an extreme degree”) of ED attitudes and behaviors. Most of the questions capture data from the past 28 days only, with exception of the ten diagnostic items that extend to the previous three months to reflect the time frame evaluated to make the *DSM* ED diagnoses. The EDE includes four subscales: Restraint (5 items), Eating Concern (5 items), Shape Concern (8 items), and Weight Concern (5 items). The subscales are averaged to give a rating of global severity. Although these subscales have not been supported in a prior factor analysis, they remain widely used in both research and clinical practice [18].

#### Parent Eating Disorder Examination (PEDE)

The PEDE version 1.4 [24] includes items that directly mirror the content and 7-point scoring scheme of the EDE. While the term “parent” is used, this measure is appropriate to use with any adult who is in the primary caretaking role. In the parent version, endorsement or denial (depending on the item) of a stem question triggers additional queries about behavioral observations and indicators of intent that are not present in the patient-directed EDE. Two additional items were added to the PEDE to assess for *refusal to maintain a normal body weight* and *denial of the seriousness of low body weight*, diagnostic features of AN that are not explicitly asked in the EDE. The item *reaction to prescribed weighing* from the EDE Weight Concern subscale was excluded because the item proved confusing when piloted. In total, the PEDE has 41 scored items. A symptom is rated as present if the parent has directly observed the phenomenon; heard the child report it; or heard reports from a reliable third party such as other family members, friends, or school personnel.

The PEDE requires that parents use their best judgment, including all available sources of information, in responding to the items. For example, in assessing *fear of weight gain*, there is not only an item evaluating verbal expression of this fear but also subsequent items assessing for indications that the young person is refusing attempts to increase their weight “by passive resistance (e.g., refusing to eat) and/or active resistance, such as yelling, throwing a tantrum, throwing food or dishes, running away, threatening to hurt themself if made to eat,” or other means. Other examples include specific questions that evaluate evidence of purging behaviors (e.g., “Have you noticed any vomit residue or odor in the bathroom or on your child’s clothes?; “Has your child rushed to the bathroom during a meal or immediately after eating?”).

The PEDE version 1.4 was developed from the EDE version 16.0 [22] and contains diagnostic items consistent with *DSM-IV-TR* [35] diagnostic criteria. Additionally, the PEDE items that assess for behavioral indicators allow for the evaluation of the revised *DSM-5* criteria, including those criteria that are not explicitly assessed by the EDE version 16.0 or 17.0 (i.e., *refusal to maintain a normal body weight* and *denial of the seriousness of low body weight*). The PEDE version 2.0 has since been revised aligning the measure with *DSM-5* diagnostic criteria and incorporating gender-neutral language, and is publicly available [36].

### Statistical Analyses

Cronbach’s alpha coefficients were calculated to evaluate the internal consistency of the EDE and PEDE subscales and global scores using IBM SPSS Statistics v.24.0, with values less than .5 considered to be unacceptable, greater than or equal to .5 poor, greater than or equal to .6 questionable, greater than or equal to .7 acceptable, greater than or equal to .8 good, and greater than or equal to .9 excellent [37].

Convergent validity was assessed through the correlation and level of agreement between the EDE and PEDE subscales. Specifically, bivariate Pearson correlations were calculated using IBM SPSS Statistics v.24.0; as suggested by Cohen [38], .10 was considered a weak or small correlation, .30 medium, and .50 or larger strong or large. Additionally, the level of agreement between the EDE and PEDE subscales and global scores was measured using a two-way random effects model (absolute agreement, average measures) intraclass correlation coefficient (ICC). In accordance with the 95% confidence interval of the ICC estimate, values less than .50 were considered evidence of poor agreement, between .50 and .75 moderate agreement, between .75 and .90 good agreement, and greater than .90 excellent agreement [39].

To assess the goodness of fit of the original four-factor structure of the traditional EDE subscales developed by Fairburn and colleagues [27], confirmatory factor analysis (CFA) was conducted with Mplus (version 8.0) [40]. Model fit was evaluated using incremental fit tests of a “good fit” [41, 42], including the Tucker-Lewis index (TLI) ≥ .90 and comparative fit index (CFI) ≥ .90. Two absolute measures of fit were also used: the standard root mean square residual (SRMR) ≤ .08 and root mean square error of approximation (RMSEA) ≤ .10 (< .05 preferred). The same procedure was repeated with the PEDE. Given the results of the CFA, an exploratory factor analysis (EFA) was conducted using IBM SPSS Statistics v.24.0 to determine if an alternate model was a better fit for the PEDE.

Planned analyses for diagnostic agreement between the PEDE and EDE included chi-squared tests and Cohen’s kappa to compare each measure’s diagnostic items.

## Results

### Internal Consistency

The coefficient alpha values for the four established subscales and global score of the EDE in the present sample ranged from acceptable to excellent: .86 for the Restraint scale, .75 for Eating Concern, .93 for Shape Concern, .83 for Weight Concern, and .93 for the global score. While the PEDE reliability coefficients for the Shape Concern and Weight Concern subscales (.85 and .74, respectively) and the global score (.80) fell in the acceptable to good ranges, alpha coefficients were poor (.59) for the Restraint subscale and unacceptable (.44) for the Eating Concern subscale.

### Construct validity

Table 1 shows the results of the Pearson correlations. There were significant medium-sized positive correlations between the corresponding subscales and global scores (all *p* values < .001) ranging from .36 to .49. In each case, the correlation with the corresponding scale of the other instrument was higher than that with any other scale. Estimates of inter-rater agreement between the EDE and PEDE subscale and global scores are shown in Table 2. There was moderate agreement between the PEDE and EDE global scores and the Restraint, Shape Concern, and Weight Concern subscale scores, and poor agreement between the Eating Concern subscales.

**Table 1:** Summary of Pearson Correlations Between Original Subscales on the EDE and PEDE

	EDE									
**PEDE**	Restraint	*n*	Eating Concern	*n*	Shape Concern	*n*	Weight Concern	*n*	Global Score	*n*
Restraint	.36^*^	369	.32^*^	369	.29^*^	369	.24^*^	368	.33^*^	369
Eating Concern	.37^*^	368	.38^*^	368	.33^*^	368	.29^*^	367	.37^*^	368
Shape Concern	.39^*^	359	.36^*^	359	.43^*^	359	.38^*^	358	.42^*^	359
Weight Concern	.39^*^	363	.35^*^	363	.42^*^	363	.42^*^	362	.43^*^	363
Global Score	.48^*^	368	.44^*^	368	.47^*^	368	.43^*^	367	.49^*^	368

Note. EDE = Eating Disorder Examination; PEDE = Parent Eating Disorder Examination

* *p* < .001, two-tailed

**Table 2:** Estimates of Inter-rater Agreement Between EDE and PEDE

Scale	*n*	ICC	*p*	95% CI
Restraint	369	.50	< .001	[.38, .59]
Eating Concern	368	.49	< .001	[.33, .60]
Shape Concern	359	.56	< .001	[.43, .66]
Weight Concern	362	.57	< .001	[.46, .66]
Global Score	368	.61	< .001	[.52, .69]

Note. EDE = Eating Disorder Examination; PEDE = Parent Eating Disorder Examination; ICC = intraclass correlation coefficient; CI = confidence interval

The CFA for the EDE four-factor model, based on established subscales, approached an acceptable fit after removing the *preoccupation with shape or weight* item from the Weight Concern factor because of a negative loading (see Table 3 for standardized factor loadings): CFI = .90, TLI = .88, RMSEA = .09, and SRMR = .05. The CFA of the four-factor model for the PEDE provided a poor fit to the data: CFI = .70, TLI = .66, RMSEA = .11 (*SE* = .10, .11); and SRMR = .10.

**Table 3:** Factor Loadings of PEDE and EDE Items

	PEDE Revised Subscales (*n* = 286)			EDE Original Subscales (*n* = 448)			
Measure Item	F1	F2	F3	Restraint	Eating Concern	Shape Concern	Weight Concern
**Restraint**							
Restraint over eating	.02	**.43**	.21	**.89**			
Avoidance of eating	.01	.14	.26	**.55**			
Empty stomach	**.43**	− .03	.07	**.81**			
Food avoidance	− .19	**.56**	.18	**.70**			
Dietary rules	− .18	**.68**	.03	**.81**			
**Eating Concern**							
Preoccupation with food	− .10	**.54**	.02		**.71**		
Fear loss of control	**.39**	.17	− .13		**.64**		
Social eating	− .03	.09	**.44**		**.61**		
Secret eating	− .19	.07	**.31**		.22		
Guilt about eating	**.37**	.24	.06		**.79**		
**Shape Concern**							
Dissatisfaction with shape	**.46**	.12	.38			**.85**	
Preoccupation with shape or weight^†^	**.55**	.23	− .16			**.71**	
Importance of shape	.24	**.62**	− .07			**.68**	
Fear of weight gain	**.56**	.29	.00			**.81**	
Discomfort seeing body	.32	− .04	**.33**			**.82**	
Discomfort about exposure	.08	− .06	**.83**			**.76**	
Feelings of fatness	**.92**	− .11	.02			**.88**	
Flat stomach	**.77**	− .08	.00			**.74**	
**Weight Concern**							
Dissatisfaction with weight	**.86**	− .15	− .09				**.83**
Desire to lose weight	**.84**	− .17	− .02				**.84**
Reaction to prescribed weighing							**.52**
Importance of weight	.28	**.54**	− .11				**.67**
Eigenvalue	6.42	1.92	1.69				
Percent of variance	3.56	9.13	8.04				
Cronbach’s alpha	.87	.75	.58				
**Correlations**							
F1							
F2	.53						
F3	.41	.20					
Restraint							
Eating Concern				.88			
Weight Concern				.81	.86		
Shape Concern				.84	.90	.94	

Note. PEDE = Parent Eating Disorder Examination; EDE = Eating Disorder Examination. Loadings from PEDE pattern matrix and from EDE Mplus standardized factor loadings. F1 = affective preoccupation with shape, weight, and eating; F2 = importance of shape, weight, and restriction; F3 = discomfort with eating and body display. Loadings in bold are > .30

^†^ Item is traditionally included in the Weight Concern subscale as well

For the EFA, the scree plot, parallel analysis, and Velicer’s minimum average partial (MAP) tests were conducted, with the latter two based on SPSS macros developed by O’connor [43]. All three tests supported retaining a three-factor model for the PEDE. Principal axis factoring (PAF) and promax rotation (power = 4) were used to extract the three factors. Loadings above .30 were used as evidence of a meaningful relationship between an item and a factor [44]. These three factors accounted for 47.7% of the total variance of the items; see Table 3. One item, *avoidance of eating*, was not associated with any scale due to insufficient loading. Looking at the items within each factor, they could be labeled as *affective preoccupation with shape*,* weight*,* and eating* (10 items, *α* = 0.87, 30.6% of total variance), *importance of shape*,* weight*,* and restriction* (7 items, *α* = 0.75, 9.1% of total variance), and *discomfort with eating and body display* (4 items, *α* = 0.58, 8.0% of total variance).

### Diagnostic Agreement

We initially planned to assess diagnostic agreement between the PEDE and EDE using chi-squared tests and Cohen’s kappa to compare each measure’s diagnostic items. However, of those participants who were not missing any EDE diagnostic items (*n* = 361), only 237 had no missing PEDE diagnostic items. A *t*-test comparison of those with and without missing PEDE diagnostic items found that participants without missing PEDE data had significantly higher PEDE global scores (*p* = .002) and significantly lower BMIs (*p* = .013) than participants who were missing PEDE diagnostic items. As the patients who could be included in this analysis appeared to have a more severe ED presentation than the remainder of the sample, results from a PEDE-EDE diagnostic comparison would be difficult to interpret. This confound precluded our conducting the planned analyses to assess diagnostic agreement.

## Discussion

To our knowledge, the PEDE is the first semi-structured interview formally developed with the aim to improve ED assessment in youth through the addition of caregiver perspectives, helping to reduce Type II error rate and under-identification of symptoms in youth with ED [45]. This study investigated the psychometric properties of the PEDE in a relatively large, international, multisite sample of families seeking treatment for AN and SAN. As predicted, the internal consistency of the PEDE was within the range of what has been published for the EDE (.44 to .85) [26], though lower than the EDE’s reliability in this sample. Regarding convergent validity, effect sizes were larger than the expected small effect size based on the meta-analytic evidence for parent-child correlation for both internalizing (.26) and externalizing (.32) disorders [6]. However, the lack of strong concordance between the EDE and PEDE subscales indicates that the information captured by the PEDE is not redundant with the EDE. This finding suggests that information from parent informants complements diagnostic and clinical information over and above that obtained by youth self-report. Specifically, the behavioral indicators and examples provided by the PEDE appear to elicit diagnostically relevant information from parents that might otherwise remain unreported. In clinical practice, such questioning can also serve to educate parents that these behaviors and beliefs are part of the ED and thereby improve their capacity to clinically monitor and intervene to support their child’s recovery.

The EDE is used with four subscales, yet none of the three studies that have examined the factor structure has replicated the four-factor model [28–30]. In this sample, the original factor structure approached an acceptable fit with the youth self-report data, but only after removing the *preoccupation with weight and shape* item from the Weight Concern subscale. Given the inconsistency of factor analysis results across studies of the EDE [26], it was not surprising that another underlying structure of three subscales seemed to provide the best fit for the PEDE. Although the PEDE has an empirically derived, three-factor structure, the original four-subscale model of the PEDE was found to measure constructs similar to those measured by the corresponding EDE scales, based on significant positive associations between corresponding subscales on the youth and parent interview. As such, it is reasonable to utilize the PEDE based on the four-subscale model to maintain consistency for research purposes. When using it for exclusively clinical purposes, the three-factor model may provide more meaningful constructs. Prior research also suggests that the EDE global score is a more useful measure of ED pathology than its subscales [46]; in light of the current study’s internal consistency and construct validity results, the PEDE global score may also provide a more valid interpretation of its findings.

Limitations of this study include a predominantly female, treatment-seeking sample with specific criteria applied, including the use of population norms (i.e., %mBMI) to determine weight eligibility instead of individualized weight status based on historical growth patterns. These limitations constrained an understanding of how the PEDE interview may perform in more diverse, transdiagnostic (including atypical AN), and non-treatment-seeking samples. Resource limitations prevented duplicate assessments by multiple interviewers to establish inter-rater reliability or compare ratings from caregivers of different genders, but this is worthy of future study, as is test-retest reliability. Furthermore, missing data precluded completion of the diagnostic agreement analyses originally proposed by this study. Although the intent of developing the PEDE was to aid in the identification of AN/SAN, future research should aim to evaluate the measure’s ability to distinguish between transdiagnostic ED cases and non-cases (i.e., criterion validity) as compared to the EDE using samples of adolescents with ED, subsyndromal ED, and no ED, and sensitivity and specificity analyses such as receiver-operator characteristic (ROC) curves. Additional work is also needed to more thoroughly assess the PEDE’s validity and predictive power, including its relationships with other measures of ED and non-ED symptoms, other parent-report measures, clinician-assigned diagnosis, and clinical outcomes. Finally, by applying more sophisticated multi-informant statistical methods [21], future research could establish how clinicians and researchers systematically integrate potentially conflicting perspectives from youth and their caregivers,.

## Conclusion

In summary, the use of parental informants is consistent with the approach to assessment of other areas of psychopathology in youth in which collateral informants frequently aid in the evaluation and diagnosis process [6, 20, 47]. The introduction of the PEDE allows for a standardized way to incorporate caregiver reports to aid in the assessment of AN, potentially reducing diagnostic ambiguity and compensating for the denial and minimization inherent in the self-report of symptoms within the group. Our future research will focus on differences in diagnostic rates when parents are enlisted as informants in interview-based AN case identification efforts. Enhanced assessment approaches can theoretically make identification of clinically significant presentations more efficient and accurate, and lead to earlier intervention and improved outcomes.

## Data Availability

The datasets used and/or analyzed during the current study are available from the corresponding author on reasonable request. The PEDE 2.0 is available at https://ccebt.com/wp-content/uploads/2024/06/PEDE-2.0_gender-neutral.pdf .

## Abbreviations

BMI: Body mass index
CFA: Confirmatory factor analysis
CFI: Comparative fit index
DSM: Diagnostic and Statistical Manual of Mental Disorders
EBW: Expected body weight
ED: Eating disorders
EDE: Eating Disorder Examination
EFA: Exploratory factor analysis
ICC: Intraclass correlation coefficient
MAP: Minimum average partial
mBMI: Median body mass index
OSFED: Other specified feeding and eating disorder
PAF: Principal axis factoring
PEDE: Parent Eating Disorder Examination
RMSEA: Root mean square error of approximation
SAN: Subsyndromal anorexia nervosa
SRMR: Standard root mean square residual
TLI: Tucker-Lewis index
US: United States
